# Insulinoma Unmasked: A Continuous Glucose Monitoring-Fueled Journey

**DOI:** 10.3390/curroncol31090403

**Published:** 2024-09-14

**Authors:** Andrijana Koceva, Mitja Krajnc

**Affiliations:** 1 Department of Endocrinology and Diabetology, University Medical Center Maribor, Ljubljanska Ulica 5, 2000 Maribor, Slovenia; 2 Faculty of Medicine, University of Maribor, Taborska Ulica 8, 2000 Maribor, Slovenia

**Keywords:** insulinoma, hypoglycemia, continuous glucose monitoring

## Abstract

Insulinomas are rare functional neuroendocrine tumors that are usually indolent and small. Due to their rarity, there is often a delay in disease recognition and diagnosis, and small tumor size makes their localization challenging. Glucose monitoring and dietary modification with or without pharmacotherapy are crucial during diagnostics, and surgery is the only definite treatment. Continuous glucose monitoring (CGM) systems can be a valuable tool in managing insulinoma patients. We present three patients with confirmed endogenous hyperinsulinemic hypoglycemia undergoing tumor localization, medical treatment, and surgery while wearing a CGM system. By accurately depicting glucose fluctuations, CGM can help prevent hypoglycemia, decrease hypoglycemia unawareness, track hypoglycemia frequency, aid in medical therapy dose titration, and confirm a cure after surgery.

## 1. Introduction

Insulinomas are rare functional neuroendocrine tumors with autonomic insulin secretion and an annual incidence of 1–4 per million [1,2,3,4]. Although rare, insulinoma is the most prevalent type of functional pancreatic neuroendocrine tumor [5]. Endogenous hyperinsulinemic hypoglycemia is the cornerstone of insulinoma clinical presentation [4]. The necessity of frequent glucose monitoring, especially in cases of hypoglycemia unawareness, can make the diagnostic period and treatment of patients with insulinoma challenging. We present a case series of three patients with confirmed endogenous hyperinsulinemic hypoglycemia undergoing tumor localization, pharmacotherapy, and surgical treatment while wearing a continuous glucose monitoring (CGM) system. We also reviewed articles indexed on PubMed over the last ten years where CGM was used for patients with insulinoma. We aim to show the efficacy of CGM in preventing hypoglycemia and managing insulinoma treatment, especially in the case of hypoglycemia unawareness.

## 2. Case Series Presentation

### 2.1. Patient 1

A 30-year-old female presented to the emergency department (ED) with an episode of disorientation and confusion at her workplace. Her glucose levels confirmed severe hypoglycemia (1.6 mmol/L), and her symptoms improved quickly after parenteral glucose administration. She reported having similar episodes in the last 6 months, particularly during fasting periods. She also gained weight due to feelings of hunger. The patient had no history of taking antihyperglycemic medication, and her past medical history, apart from polycystic ovary syndrome, was uneventful. A supervised fast was performed and the test was stopped after 28 h due to symptoms of confusion and disorientation, with a glucose level of 1.3 mmol/L, insulin levels of 46.48 mU/L (reference range 2.6–24.9 mU/L), and C-peptide of 1.21 nmol/L (reference range 0.27–1.27), confirming endogenous hyperinsulinemic hypoglycemia. She was fitted with a CGM sensor Dexcom G6 (Dexcom, Inc. San Diego, CA, USA). The percentages of sensor glucose concentrations or time below range (TBR) of <3.9 mmol/L and <3 mmol/L were 8% and 4%, respectively. With further CGM use alongside diet modification, the TBR decreased to 4% and <1% and after diazoxide initiation to 1% and <1%, respectively. She underwent an abdominal and thoracic computed tomography (CT) scan as well as an abdominal magnetic resonance imaging (MRI) with a cholangiopancreatography (MRCP), which revealed a 2 cm hyperintense hypervascular tumor at the body of the pancreas in close contact with the main pancreatic duct. An R0 resection with a left pancreatectomy was conducted, and histology confirmed a 1.4 × 1.5 cm well-differentiated neuroendocrine pancreatic tumor (grade 1) with a Ki67 of 1.5% and less than 1 mitosis/2 mm^2^. CGM readings after surgery showed transient hyperglycemia followed by normal glucose levels and a TBR of 0%, confirming a surgical cure.

### 2.2. Patient 2

A 50-year-old man was taken to the ED after displaying violent behavior and confusion while driving. His wife gave him chocolate and called an ambulance. His point-of-care blood glucose was 3.2 mmol/L, which normalized after parenteral glucose administration, and his symptoms were relieved. He reported having a similar incident of aggression with amnesia two years ago, for which he was examined in the psychiatric clinic and was diagnosed with dissociative amnesia and dissociative motor disorder. A supervised fast was conducted and terminated after 7 h due to the onset of retrograde amnesia, with a glucose level of 1.5 mmol/L, an insulin level of 74.97 mU/L (reference range 2.6–24.9 mU/L), and a C-peptide of 1.37 nmol/L (reference range 0.27–1.27), confirming endogenous hyperinsulinemic hypoglycemia. Because of his hypoglycemia unawareness, he was equipped with a CGM Dexcom G6 (Dexcom, Inc. San Diego, CA, USA). Initial TBRs were 6% (<3.9 mmol/L) and 1% (<3 mmol/L). He was advised on dietary interventions and later started on diazoxide, the dose of which was adjusted based on his CGM readings to 300 mg per day during the diagnostic workup. A contrast-enhanced abdominal CT scan and an abdominal MRI with MRCP revealed a 1.6 × 1.3 cm hypervascular pancreatic tumor in the head–neck transition. Upon follow-up, he was feeling better and more energized, and his CGM readings showed fewer hypoglycemic episodes with a TBR of 2% in the range of <3.9 mmol/L and <1% in the range of <3 mmol/L. Although diazoxide dose titration was stopped due to peripheral swelling, the patient felt safer knowing he had a CGM system with an alarm function and continued successfully mitigating hypoglycemia. The TBR remained at 2% in the range of <3.9 mmol/L and <1% in the range of < 3 mmol/L. The patient underwent tumor enucleation, and histology showed a 1.7 cm well-differentiated neuroendocrine pancreatic tumor (grade 1) without vascular or perineural invasion with up to 2 mitoses/2 mm^2^ and a Ki67 of 1.8%. Following surgery, CGM recorded transient postoperative hyperglycemia which was followed by normalization of glucose levels with a TBR of 0%.

### 2.3. Patient 3

A 28-year-old female was seen at the endocrinology outpatient clinic complaining of episodes of hunger and dizziness, tremors, palpitations, and sweating that were relieved after eating. During one of these episodes, she underwent a finger-prick glucose measurement that showed a glucose level of 3 mmol/L. She had these occasional episodes for a year, but they became more frequent and often occurred at night. Her past medical history was unremarkable, and she was not taking any antihyperglycemic drugs. A supervised fast was performed and terminated after 3 h with symptoms of hypoglycemia, a glucose level of 2.7 mmol/L, an insulin level of 10.7 mU/L (reference range 2.6–24.9 mU/L), and a C-peptide of 0.32 nmol/L (reference range 0.27–1.27), confirming endogenous hyperinsulinemic hypoglycemia. An abdominal contrast-enhanced MRI and endoscopic ultrasound (EUS) were conducted, but no pancreatic lesion was found. She then underwent a Ga-68 DOTATATE PET/CT at the Nuclear Medicine Department, UMC Ljubljana, revealing a small focal enhancement of SST receptors on the upper border of the pancreatic body with no visible lesion on the CT scan. A second EUS showed a suspected small 7.4 × 6.4 mm hypoechogenic change in the pancreatic parenchyma at the body–tail transition. After three months, EUS was then repeated for the third time and showed the same slight inhomogeneity at the pancreatic body–tail transition, without an evident tumor. Despite dietary interventions and self-monitoring of blood glucose (SMBG), she had another severe hypoglycemia with neuroglycopenic symptoms for which she was seen at the ED, so she was equipped with a CGM sensor Dexcom G6 and started diazoxide with a total daily dose of 225 mg. Initial CGM readings showed a TBR of 21% in the range of <3.9 mmol/L and 2% in the range of <3 mmol/L, which later decreased to 3% and <1%, respectively. Finally, 9 months after biochemical diagnosis, a second abdominal MRI with MRCP confirmed a 1.7 × 1.0 cm hypervascular tumor at the pancreatic body–tail transition beside the main pancreatic duct. A left pancreatectomy was conducted, and histology showed a 1.1 cm well-differentiated neuroendocrine tumor (grade 1–2) with up to 2 mitoses/2 mm^2^ and a Ki67 of 6%. Following surgery, CGM surveillance confirmed a surgical cure with the TBR being 0%.

The results of the supervised fasting test as well as changes in the measured percentage of the TBR during different stages of treatment in all patients are summarized in Table 1.

## 3. Discussion

Insulinomas are usually solitary tumors, with equal distribution throughout the head, body, and tail of the pancreas. They are rarely multifocal, and 3% of cases are even ectopic, with the duodenal mucosa being the most common location [6]. Despite typically being indolent tumors, in 10 to 15% of cases, insulinomas can display aggressive or malignant tumor behavior [1,4]. While most are sporadic, approximately 5–10% may appear in hereditary syndromes such as multiple endocrine neoplasia type 1 (MEN 1) [1,7]. Insulinomas in the setting of MEN-1 manifest earlier, at a younger age than the sporadic forms, tend to be larger at diagnosis, and have a higher likelihood of being multifocal [8]. The incidence of insulinoma recurrence is also higher in those appearing in the setting of MEN-1 syndrome, with a 10-year recurrence rate of 21% in contrast to 5% in the sporadic cases [3]. Indolent insulinomas have a reported 5-year survival rate of 94.5–100%, in contrast to 24–66.8% in aggressive insulinomas [4,9,10]. In our case series, all three patients displayed a well-differentiated small indolent insulinoma, less than 2 cm in size. Due to their young age at presentation, genetic testing was conducted in patients 1 and 3, with the results yet to be received.

Endogenous symptomatic hyperinsulinemic hypoglycemia is the cornerstone of the insulinoma clinical presentation. The Whipple triad, which consists of symptoms suggestive of hypoglycemia, confirmation of low blood glucose concentration, and symptom alleviation upon the administration of carbohydrates, is a crucial clinical diagnostic factor for endogenous hyperinsulinemic hypoglycemia [11]. Hypoglycemia, depending on the glucose level, can present with autonomic symptoms, caused by sympathoadrenal activation, or with neuroglycopenic symptoms due to glucose deprivation in the central nervous system [12]. Autonomic symptoms include sweating, palpitations, hunger, and tremors, while neuroglycopenic symptoms encompass cognitive impairment, visual disturbances, and irritability and may progress to seizures and coma [11,13,14]. Exercise or fasting are usual precipitating factors of hypoglycemia [6]. Most patients experience exclusively fasting hypoglycemia (73%). Isolated postprandial hypoglycemia (6%) or a combination of fasting and postprandial hypoglycemia (21%) are less frequent [15]. All of our patients had mainly fasting hypoglycemia presenting with autonomic and neuroglycopenic symptoms in the case of patient 1, mostly neuroglycopenic symptoms in patient 2, and mostly autonomic symptoms in patient 3. Recurrent hypoglycemic episodes may lead to desensitization of the patient and the development of hypoglycemia unawareness, which was most evident in patient 2 but was also present in patient 1 [11,16]. Many patients with insulinoma as in the case of patient 2 are seen by multiple physicians before the diagnosis, often by a psychiatrist or a neurologist, due to neuroglycopenic symptoms masquerading as different diseases [12]. The condition, unfortunately, is not readily recognized or even thought of, and the average symptom duration before diagnosis varies from a few months to years [3,17,18,19,20], with up to 50% of patients diagnosed with insulinoma 5 years after symptom onset [21]. Patient 1 had symptoms for 6 months, patient 3 for 1 year, and patient 2 had symptoms lasting for 2 years before diagnosis.

The supervised prolonged fasting test is the gold standard for diagnosing endogenous hyperinsulinemic hypoglycemia [11,22]. The test consists of a 72 h supervised fast in an inpatient setting with ongoing monitoring of plasma glucose, insulin, and C-peptide levels with or without beta-hydroxybutyrate until hypoglycemia occurs. The test is considered positive if there is symptomatic hypoglycemia with concurrent inappropriately elevated levels of insulin (at least 3 μU/mL or 18 pmol/L), proinsulin (at least 5 pmol/L), C-peptide (at least 0.2 nmol/L), and suppressed serum beta-hydroxybutyrate (less than 2.7 mmol/L) in the absence of sulfonylurea in the plasma. A rise in glucose is typically seen after glucagon injection indicating preserved glycogen stores [11,22]. The supervised fasting test in our series was terminated after 28 h in patient 1, 7 h in patient 2, and 3 h in patient 3.

The primary imaging modalities to localize an insulinoma are multiphasic abdominal contrast-enhanced CT imaging, abdominal MRI, and/or EUS. EUS can detect even small lesions between 2 and 5 mm but can overlook lesions located at the pancreatic tail, and its success is operator-dependent [11,23]. All our patients underwent an abdominal CT and MRI scan; only patient 3 had a CT scan, two MRIs, and three EUS before localizing the insulinoma. Nuclear medicine functional imaging has also improved the localization and characterization of insulinomas. Since indolent insulinomas express somatostatin receptors (SSTRs) and glucagon-like peptide-1 receptors (GLP-1Rs), SSTR or even more sensitive GLP-1 receptor imaging can also be used [24]. Since the tumor was not visible on the first CT/MRI scans, only patient 3 underwent a 68Ga-DOTATATE PET/CT scan, which confirmed the focal enhancement of SST receptors.

All patients awaiting definitive treatment require adequate control of life-threatening hypoglycemia episodes, which can be effectively controlled by dietary modifications aimed at preventing extended periods of fasting with or without pharmacological therapy [11,23,25]. Patients should adhere to general recommendations that entail consuming complex carbohydrates with slow absorption at consistent intervals to avoid prolonged fasting and are advised to refrain from engaging in activities such as driving and excessive exercise [6,11]. Patient education on hypoglycemia recognition and treatment is crucial, and it is also advisable to offer proper education to friends and family members. [6]. In the case of severe hypoglycemia, when the patient cannot consume carbohydrates, the administration of glucagon intramuscularly or intranasally is indicated [6,11]. All patients were educated on dietary modification, hypoglycemia prevention and treatment, and glucose monitoring with SMBG as well as CGM and were prescribed glucagon.

Diazoxide, a benzothiadiazine derivative, is typically the preferred initial first-line medication for patients diagnosed with insulinoma. Diazoxide is associated with several adverse effects, including dose-dependent edema, hirsutism, gastrointestinal discomfort, nausea, and weight gain [6,25]. Somatostatin analogs (SSAs), specifically octreotide and lanreotide, have also been identified as a suitable, second-line medical treatment when diazoxide is ineffective or not tolerated or first-line medical treatment when facing a malignant insulinoma [6,11]. All three patients adhered to dietary modifications and were treated with diazoxide. Patient 2 developed ankle edema, which limited the diazoxide dose increase. Nevertheless, with the help of CGM, he effectively managed to prevent episodes of hypoglycemia, and thus second-line treatment was not initiated.

Surgery is the gold standard and definite treatment for localized insulinomas [11,23]. Various surgical procedures have been employed for insulinoma treatment, with the decision primarily based on tumor size, location, and relationship to the main pancreatic duct [11,23,25]. Since insulinomas are typically benign and minimally invasive, parenchyma spearing techniques should always be considered when technically possible, and enucleation is usually proposed for insulinomas smaller than 2–3 cm and located ≥ 3 mm from the main pancreatic duct [11,26]. All our patients underwent surgery; patients 1 and 3 had left pancreatectomy, and patient 2 had tumor enucleation. In all patients, CGM confirmed a surgical cure.

## 4. Use of CGM in Insulinoma Patients

Effective glucose monitoring in patients with insulinoma can be challenging since the traditional SMBG, even when conducted regularly, provides only intermittent plasma glucose measurements at a single point in time and therefore is less effective for assessing glucose variability, monitoring glucose patterns, quantifying hypoglycemia, or evaluating the efficacy of dietary/medical treatment. CGM systems, on the other hand, enable continuous glucose measurements [27,28]. CGM usually has three components: a wearable sensor inserted subcutaneously to measure glucose concentration in the interstitial fluid at regular intervals, a transmitter that sends readings, and a receiver or compatible smart device that displays readings to the user [29]. Daily use of a CGM provides patients with regular feedback on current glucose levels and glucose trends, including the direction and rate of change in glucose levels [27,30]. To access glucose control based on measured sensor glucose, CGM systems provide three measurements: the percentage of readings and time per day within the target glucose range (TIR) (usually between 3.9 and 10 mmol/L), time below target glucose range (TBR) (level 1 with sensor glucose < 3.9 mmol/L or level 2 with sensor glucose < 3 mmol/L), and time above target glucose range (TAR) (level 1 with sensor glucose > 10 mmol/L or level 2 with sensor glucose > 13.9 mmol/L) [30]. Many real-time CGM systems also have alarm functions that can notify the user in case of hypoglycemia, which is particularly useful when users are otherwise not aware, for example, during sleep or other activities, such as exercise [29]. Currently, with technological advances, some real-time CGM systems, such as Dexcom G6 used in our series, also have predictive alarms, that, based on glucose values and the rate of change, can predict an upcoming hypoglycemic event, enabling the user to take preventative instead of corrective measures [29]. Lastly, some CGM systems allow for real-time glucose data sharing with family members or clinicians, providing an additional layer of safety, especially in pediatric or elderly patients [28]. The use of CGM in patients with diabetes has been shown to increase the TIR, lower HbA1c, and/or reduce hypoglycemic events, as well as improve diabetes-related quality of life (i.e., decreased diabetes distress, increased hypoglycemic confidence, and reduced fear of unexpected hypoglycemia) [27,31,32,33,34,35,36,37]. Although the existing body of the literature predominantly focuses on utilizing CGM in the context of diabetes care, with the technological advancements and CGM accessibility, there is a growing interest in exploring additional applications of CGM, such as the use of CGM in patients with insulinoma. For the past 10 years, numerous case reports and some studies have documented the use of CGM among patients with insulinoma, demonstrating its utility in hypoglycemia prevention, improving diagnostics, and analyzing glycemic outcomes during different treatment modalities.

CGM has been used for screening and insulinoma identification as well as for confirming and preventing hypoglycemia during the diagnostic period. Gu et al. used CGM to analyze different characteristics of glucose metabolism indexes in 22 patients with insulinoma and suggested that some indexes, such as CONGA (continuous overall net glycemic action), M value, and LBGI (low blood glucose index) might be used for insulinoma identification [38]. In a single-center prospective observational case–control study, Ma et al. also used CGM to screen patients for insulinoma and showed that CGM glucose patterns differ between patients with insulinoma and functional hypoglycemia. The group additionally proposed that the coefficient of variation (CV) can help identify insulinoma etiology and a CV of 19% was found to be effective for the screening of insulinoma in the outpatient setting [39]. Maher et al. used Dexcom G6 to confirm exclusively postprandial hypoglycemia and a surgical cure in a patient with a metastatic proinsulin-secreting NET [40]. Tomazic et al. used Medtronic MiniMed CGM to confirm hypoglycemia in a pregnant female with insulinoma [41]. Suzuki et al. showed the utility of CGM in confirming the presence of nocturnal hypoglycemia in patients presenting with abnormal nocturnal behavior. These patients were initially suspected to have rapid eye movement sleep behavior disorder (RBD) or epilepsy but were later successfully diagnosed with an insulinoma [42]. Similarly, Haba et al. showed a case of a patient presenting with neuropsychiatric symptoms who was initially misdiagnosed with schizophrenia. CGM use helped confirm hypoglycemia and led to the diagnosis of insulinoma [43].

CGM has also been used to evaluate medical treatment efficacy and the need for dose adjustments. Vezzosi et al. used CGM in 27 patients with endogenous hypoglycemia receiving pharmacological treatment with diazoxide, SSA, or glucocorticoids [44]. Although these patients were initially considered normoglycemic, as they were asymptomatic and had normal SMBG values, CGM recordings showed that 56% of these patients experienced asymptomatic hypoglycemia and/or hyperglycemia requiring additional treatment modification, confirming the utility of CGM in the identification of otherwise asymptomatic glycemic excursions [44]. Aida and Noto used FreeStyle Libre Pro on a patient with insulinoma for diazoxide dose adjustments and to confirm a surgical cure [45]. Suminaga et al. used the same system for a patient requiring continuous glucose infusion besides oral diazoxide treatment [46]. They also compared CGM glucose values with capillary blood glucose (CBG) values of two insulinoma patients and examined mean absolute relative differences (MARDs) and absolute glucose differences in several conditions (during fasting, postprandially, and with hypoglycemia). CGM values were mainly in accordance with CBG values and MARDs, and absolute glucose difference values were found to be acceptable [46]. Murakami et al. used CGM to confirm hypoglycemia and evaluate medical and surgical treatment in a patient with a history of epilepsy presenting with hypoglycemia-induced seizures [47]. Manaka et al. used the CGM system to evaluate treatment with glucose infusion, diazoxide, or lanreotide in an elderly patient with insulinoma and showed that lanreotide is a useful alternative in the case of diazoxide side effects [48]. Yanagiya et al. used CGM to evaluate everolimus efficacy in a patient with inoperable metastatic insulinoma [49]. CGM has also been used to assess glucose control and evaluate medical treatment in patients with insulinoma and concomitant diabetes [50,51] as well as to evaluate the efficacy of EUS-guided ethanol injection treatment in eight patients who were unable or refused to undergo surgery [52].

CGM use has also been reported in patients awaiting surgery, during insulinoma surgery, and during postoperative follow-up. Sugawa et al. used FreeStyle Libre Pro to prevent severe hypoglycemia while awaiting surgery in a patient with hypoglycemia unawareness who refused pharmacotherapy [53]. Sugiyama et al. used an Enlite sensor MiniMed 620G in combination with intermittent blood glucose measurements to monitor glucose trends and adjust glucose infusion during insulinoma surgery [54]. CGM has also been used in patients with insulinoma before and after surgery to confirm the occurrence of reactive hypoglycemia in the preoperative period [55] and the lowering of the TBR after surgery [56]. As a third of patients with insulinoma can develop rebound hyperglycemia after insulinoma surgery, some advise close glucose monitoring in the first 24 h after surgical treatment; this rebound hyperglycemia can also be objectively quantified and mitigated using real-time CGM [57,58]. CGM has been used even during an 8-year follow-up and active surveillance of a patient with recurrent malignant duodenal insulinoma and lymph node metastases treated surgically [59].

Our case series shows the utility of CGM in three patients diagnosed with insulinoma during all stages of management, starting from diagnostics to non-pharmacological, pharmacological, and surgical treatment, as well as during the postoperative period. All patients had high TBRs in their initial CGM readings. Further CGM use alongside diet modification and diazoxide treatment led to an increase in the TIR and a decrease in the TBR at both levels. Similarly, as previously reported [57], CGM surveillance after surgery showed a transient rebound hyperglycemia in patients 1 and 2, followed by normalization of glucose values and a TBR of 0%, which confirmed surgical cure in all patients. With this case series, we show that CGM can be a valuable tool for preventing hypoglycemia, monitoring hypoglycemia frequency and duration during dietary and pharmacological treatment, improving the management of hypoglycemia unawareness, evaluating medical treatment efficacy and the need for dose adjustments, and confirming a surgical cure.

## 5. Conclusions

Insulinoma is a rare functional neuroendocrine tumor causing endogenous hyperinsulinemic hypoglycemia. It manifests with various signs and symptoms, depending on tumor size, insulin levels, and disease duration. Due to its rarity, there is usually a diagnostic delay. Good tumor localization is crucial for successful surgical tumor resection, and glucose control and monitoring during diagnostics can be challenging. Continuous glucose monitoring can be a valuable tool in managing insulinoma patients. Promptly alerting patients of upcoming hypoglycemia and the need for additional snacks aids in preventing hypoglycemia, and it is especially valuable for patients with hypoglycemia unawareness. In our experience, CGM can be beneficial in patients with insulinoma as a valuable tool for preventing hypoglycemia, monitoring medical therapy success, and confirming a cure after surgery.

## Figures and Tables

**Table 1:** Laboratory data during the 72 h fast and TBR during different treatment modalities.

		Patient 1	Patient 2	Patient 3
Fasting time (h)		28	7	3
Plasma glucose (mmol/L)		1.3	1.5	2.7
Insulin levels (mU/L)		46.48	74.97	10.7
C-peptide (nmol/L)		1.21	1.37	0.32
Initial TBR (%)	3.0–3.8 mmol/L	8%	6%	21%
	<3 mmol/L	4%	1%	2%
TBR during diet modification (%)	3.0–3.8 mmol/L	4%	2%	/
	<3 mmol/L	1%	<1%	/
TBR during diazoxide (%)	3.0–3.8 mmol/L	1%	2%	3%
	<3 mmol/L	<1%	<1%	<1%
TBR after surgery (%)	3.0–3.8 mmol/L	0%	0%	0%
	<3 mmol/L	0%	0%	0%

## Data Availability

Supporting data can be provided by the authors upon a reasonable request.
